# Cultural Diffusion Was the Main Driving Mechanism of the Neolithic Transition in Southern Africa

**DOI:** 10.1371/journal.pone.0113672

**Published:** 2014-12-17

**Authors:** Antonieta Jerardino, Joaquim Fort, Neus Isern, Bernardo Rondelli

**Affiliations:** 1 ICREA/Department of Experimental & Health Sciences, Universitat Pompeu Fabra, CaSEs Research Group, Ramon Trias Fargas 25-27, 08005 Barcelona, Spain; 2 Complex Systems Lab, Department of Physics, University of Girona, C/. M^a^ Aurèlia Capmany 61, 17071 Girona, Catalonia, Spain; 3 Laboratori d'Arqueologia Quantitativa (LAQU), Departament de Prehistòria, Universitat Autònoma de Barcelona, 08193 Cerdanyola del Vallès, Spain; 4 CaSEs Research Group, Department of Archaeology and Anthropology, Institució Milà i Fontanals, Spanish National Research Council (IMF-CSIC), C/Egipcíaques, 15, 08001 Barcelona, Spain; Universidade do Algarve, Portugal

## Abstract

It is well known that the Neolithic transition spread across Europe at a speed of about 1 km/yr. This result has been previously interpreted as a range expansion of the Neolithic driven mainly by demic diffusion (whereas cultural diffusion played a secondary role). However, a long-standing problem is whether this value (1 km/yr) and its interpretation (mainly demic diffusion) are characteristic only of Europe or universal (i.e. intrinsic features of Neolithic transitions all over the world). So far Neolithic spread rates outside Europe have been barely measured, and Neolithic spread rates substantially faster than 1 km/yr have not been previously reported. Here we show that the transition from hunting and gathering into herding in southern Africa spread at a rate of about 2.4 km/yr, i.e. about twice faster than the European Neolithic transition. Thus the value 1 km/yr is not a universal feature of Neolithic transitions in the world. Resorting to a recent demic-cultural wave-of-advance model, we also find that the main mechanism at work in the southern African Neolithic spread was cultural diffusion (whereas demic diffusion played a secondary role). This is in sharp contrast to the European Neolithic. Our results further suggest that Neolithic spread rates could be mainly driven by cultural diffusion in cases where the final state of this transition is herding/pastoralism (such as in southern Africa) rather than farming and stockbreeding (as in Europe).

## Introduction

In previous work, the rate of spread of the Neolithic transition in Europe has been accurately quantified by means of linear regressions, which yield a speed of about 1 km/yr [1], [2]. The same approach has been applied to the initial peopling of America [3]. But the spread rate of the Neolithic transition in continents other than Europe has not been accurately quantified by means of sound statistical techniques (e.g., linear regressions over large distances). For this reason, at present there are two open possibilities. Perhaps a value of about 1 km/yr is characteristic only of Europe. Alternatively, if such a spread rate were observed in several continents, it could reflect an intrinsic feature of the shift from a hunting and gathering way of life into farming/herding, i.e. a universal law for Neolithic transitions all over the world. In order to answer this question, it is necessary to quantify spread rates of the Neolithic transition in continents other than Europe through the analysis of numerous, high-quality data.

There are two main models of Neolithic transitions. According to the demic model [4], the spread of Neolithic economies is due to a range expansion of Neolithic populations (i.e., farmers/herders). According to the cultural model [5], it is due to the conversion of hunter-gatherers into farmers/herders. However, some authors have argued for the importance of both demic and cultural diffusion [6], [7].

**Figure 1:**
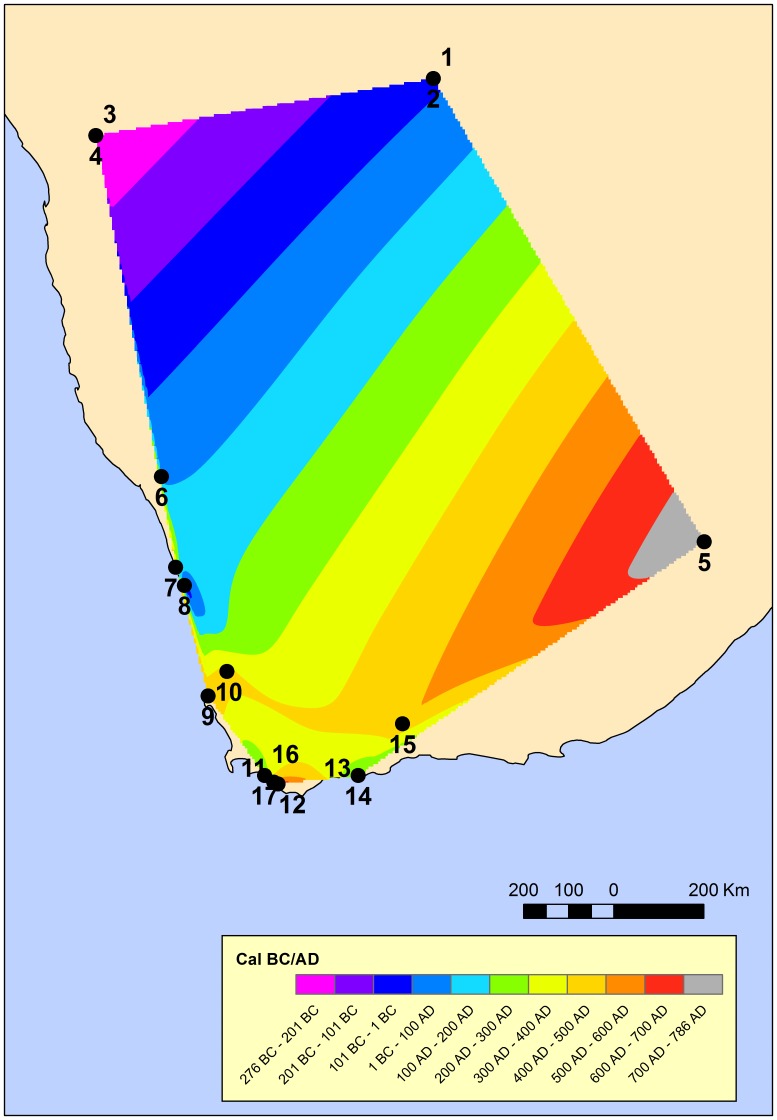
Neolithic chronology in southern Africa. Map obtained from a natural neighbor interpolation of 17 early Neolithic calibrated dates (symbols) in southern Africa. Color regions correspond to the area advanced by the Neolithic wave every 100 years. Each date is identified by a number and its details are given in table 1. All dates are in calibrated years BC/AD.

**Figure 2:**
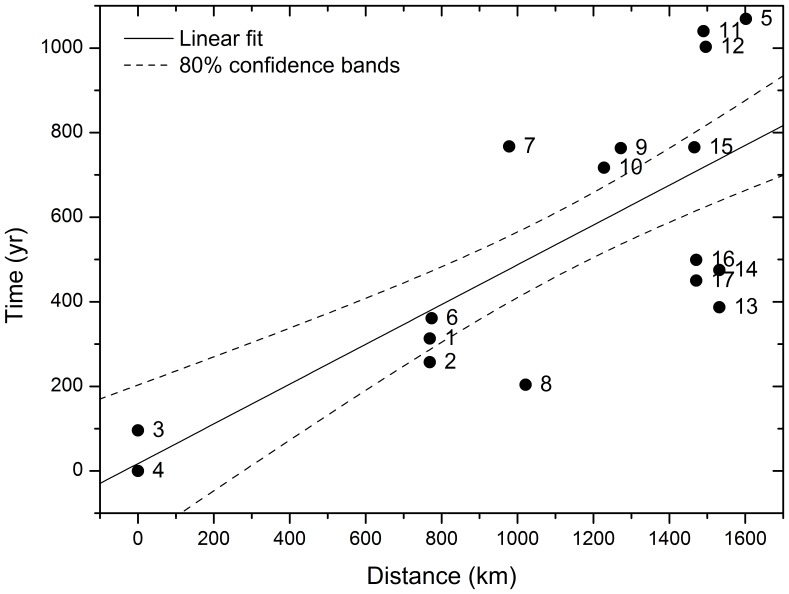
Linear regression fit to determine the speed of the Neolithic fronts using all dates and great-circle routes. Symbols correspond to the 17 dates in table 1. Time and distances are computed from the oldest site (labelled 4 in the Fig. and in table 1) and using great-circle routes and calibrated dates. The solid line corresponds to the linear regression fit and the dashed lines are the 80% confidence bands. The 80% confidence level range obtained for the front speed is 1.4–2.8 km/yr with a correlation coefficient 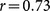.

In the European case, the observed value of about 1 km/yr [1], [2], [7] is due to the spread being driven mainly by demic diffusion (i.e., with cultural diffusion playing a secondary role) according to a recent wave-of-advance model that unifies demic and cultural diffusion [8]. Also according to that model, if additional Neolithic spread rates of about 1 km/yr were observed (in other continents), they would also indicate mainly demic diffusion transitions. In contrast, if a Neolithic spread rate were substantially faster than 1 km/yr, cultural diffusion would have a more important effect than demic diffusion [8] (see Figs. 1 and 2 therein). However, Neolithic spread rates substantially faster than 1 km/yr have not yet been observed. Here we report such a fast Neolithic spread rate, and analyze its implications on the importance of demic and cultural diffusion.

A related open problem is that, in some cases, the Neolithic transition was not a shift from hunter-gathering into farming (with stockbreeding) but into herding or fully developed pastoralism. Pastoralism is understood here as “…a way of life that was economically, socially and symbolically focused on livestock management” [9], while herding is regarded as smaller in scale and with economic flexibility that allows complementary subsistence activities such as hunting, gathering and fishing [10], [11]. Late fifteenth and early sixteenth centuries historical records from the Cape of Good Hope (present day South Africa) describe pastoralists (the ‘Khoikhoi’) who reared domestic cattle and sheep in large numbers and also possessed goats and dogs and made ceramics. These groups neither grew crops nor did they have an iron-working technology (in contrast with the Bantu Iron Age farmers who moved and settled into the eastern half of southern Africa about 2000 years ago). Non-agricultural herding spread to central, western and southern parts of southern Africa as explained below [9], [12]. There is a consensus that Khoikhoi domesticates must have been introduced from north of the Equator as they do not have wild progenitors in southern Africa [13]–[14]. More debate surrounds the origins, timing, hypothetical routes by which domesticates would have spread southwards [13], [15]–[17], and the mechanisms involved in their introduction to southern Africa [9], [10], [14], [18]–[22].

Purely demic diffusion models of the spread of herding across southern Africa assume that groups of immigrant people moved through various landscapes bringing first sheep and then cattle. Such models assume that the conversion of hunter-gatherers into herders did not have an important effect in this process, so we refer to them as purely demic models. Within purely demic models, different authors have proposed different routes for the introduction of stock [15]–[17], [23], but in the present paper we will not tackle the problem of which route was more likely. Instead, our purpose here is to contribute to the debate between demic and cultural models.

Proponents of cultural diffusion argue that stock and herding skills were initially introduced by pastoralists to neighboring hunter-gatherers who then passed on the knowledge of herding and live animals to other nearby hunter-gatherer groups through exchange networks [9], [10], [14], [18], [19], [24]. Mid- to late-twentieth-century Kalahari hunter-gatherers who kept small flocks of goats while continuing to rely on wild food [25]–[27] are often presented as analogues for past small-scale herder-forager groups responsible for the introduction of stock into southernmost Africa. The possibility of several potential episodes of small-scale demic migrations and hybridization, however, is recognized by some of those who defend a cultural diffusion model [9], [24].

Much of the evidence brought to bear in these models range from studies on linguistics, oral traditions, diversity of ceramic styles, types of rock art imagery, and on absolute dates for the earliest livestock bones and ceramics, quantified observations on other material culture recovered from archaeological excavations, and lately also genetic data [9], [20], [21], [28]–[33]. However, with the exception of the work by Russell [21] on the spread of a specific domesticate (the African fat-tailed sheep), which is discussed in the Results section below, no other study supported by mathematical modelling of spatially mapped data has been undertaken until now.

Since 2004, a number of sites with reliably dated early domestic fauna have been excavated and published (Table 1). Here we analyze these data to estimate the spread rate of this process, as necessary to calculate the relative contributions of demic and cultural diffusion in the framework of a recent wave-of-advance model [8].

**Table 1:** Early Neolithic non-Bantu radiocarbon dated domestic fauna from southern Africa.

Label	Site name	country/region	Latitude	Longitude	C14 date (uncal.) BP	Calibrated date (µ), BP	Calibrated age range (2σ), BP	Calibrated date (µ), BC/AD	Calibrated age range (2σ) BC/AD	Lab number	Domestic species present	material dated	Reference
1	Toteng 1	Botswana	−20.4481	22.8667	2020±40	1916	2032–1820	34 AD	83 BC–131 AD	Beta-186669	Sheep (Ovies aries)	Bone: right astragalus	[34]
2	Toteng 1	Botswana	−20.4481	22.8667	2070±40	1972	2111–1875	23 BC	162 BC–75 AD	Beta-1904888	Cow (Bos taurus)	Bone: 2nd and 3rd carpal, right side	[34]
3	Leopard Cave	Namibia	−21.5728	15.5550	2190±40	2133	2307–1994	184 BC	358–45 BC	Beta –270163	Caprine (Sheep/goat)	Tooth	[35]
4	Leopard Cave	Namibia	−21.5728	15.5550	2270±40	2229	2337–2129	280 BC	388–180 BC	Beta –270164	Caprine (Sheep/goat)	Tooth	[35]
5	Likoaeng	Lesotho	−29.7333	28.7500	1285±40	1160	1270–1061	790 AD	680–890 AD	GrA-23237	Sheep (Ovies aries)	Bone: right ulna	[36]
6	/Ai Tomas	South Africa (NC)	−28.4167	16.9500	1980±80	1868	2108–1633	82 AD	159 BC–317 AD	Pta-5530	Sheep (Ovies aries)	Bone (wild vertebrate)	[37], [38]
7	KN2005/041	South Africa (NC)	−30.2350	17.2500	1625±25	1462	1530–1392	488 AD	421–559 AD	OxA-22933	Cow (Bos taurus)	Horn core	[29]
8	Spoeg River Cave	South Africa (NC)	−30.6001	174 423	2105±65	2025	2299–1835	76 BC	350–115 BC	OxA-3862	Sheep (Ovies aries)	Bone: 3rd phalange	[13]
9	Kasteelberg A	South Africa (WC)	−32.8147	17.9484	1630±60	1466	1607–1338	484 AD	343–613 AD	OxA-3864	Sheep (Ovies aries)	Bone: thoracic vertebra, juvenile	[13]
10	Tortoise Cave	South Africa (WC)	−32.3270	18.3594	1680±50	1512	1691–1391	438 AD	260–559 AD	Pta-3312	Sheep (Ovies aries)	charcoal	[39]
11	Die Kelders	South Africa (SWC)	−34.5462	19.3753	1325±60	1189	1297–1062	761 AD	654–888 AD	OxA-3860	Sheep (Ovies aries)	Bone: 2nd phalange	[13]
12	Byneskranskop	South Africa (SWC)	−34.5833	19.4667	1370±60	1226	1330–1080	724 AD	620–870 AD	OxA-3863	Sheep (Ovies aries)	Bone: mandibular condyle	[13]
13	Blombos Cave	South Africa (SC)	−34.4167	21.2167	1960±50	1842	1983–1713	108 AD	34 BC–237 AD	OxA-4543	Sheep (Ovies aries)	Bone: left mandible	[40]
14	Blombos Cave	South Africa (SC)	−34.4167	21.2167	1880±55	1754	1888–1607	196 AD	62 BC–344 AD	OxA-4544	Sheep (Ovies aries)	Bone: calcaneum	[40]
15	Boomplaas	South Africa (SC)	−33.3833	22.1833	1630±50	1464	1567–1347	486 AD	384–604 AD	UW-337	Sheep (Ovies aries)	charcoal	[41]
16	Hawston	South Africa (SC)	−34.4119	19.1739	1860±60	1730	1873–1569	220 AD	77–382 AD	Pta-834	Sheep (Ovies aries)	charcoal	[42]
17	Hawston	South Africa (SC)	−34.4119	19.1739	1900±40	1779	1883–1634	171 AD	68–317 AD	Pta-835	Sheep (Ovies aries)	charcoal	[42]

All uncalibrated dates are corrected for ^13^C. Dates are calibrated using ShCal04 calibration curve for the Southern Hemisphere (43). Coordinates are expressed as decimal degrees. Regions within South Africa are indicated with acronyms: Northern Cape (NC), Western Cape (WC), South Western Cape (SWC), and Southern Cape (SC).

## Materials and Methods

### 1. Database

Table 1 shows the earliest dates published to this date for domestic fauna from 13 southern African archaeological sites. For some sites we include two dates because either it is not clear that the earliest one is directly associated with domestic animals, or because the archaeological remains are bounded by the two dates reported. From a total of 17 dates, 12 were obtained directly (by accelerator mass spectrometry) from positively identified domestic faunal remains, such as bone, teeth and horn core. One date was determined on bone of wild fauna that was securely associated with domestic fauna (Table 1, site/Ai Tomas), while 4 other dates were determined on charcoal recovered from stratigraphic layers where domestic fauna was reliably identified. Early domestic fauna consist of mostly African sheep (*Ovies aries*), some cow remains (*Bos taurus*), and possibly also goat (*Capra hircus*) (Table 1 and refs therein). We have not limited our study to sheep only, partly because this would render a smaller sample size for our statistical analyses and also because our interest is with the early herding in southern Africa without limiting it to a particular species of animal domesticate. Calibration of radiocarbon dates was done using the OxCal program (https://c14.arch.ox.ac.uk) and the ShCal04 calibration curve for the Southern Hemisphere [43]. Geographic positions of sites were obtained from published data (either coordinates or maps). No archaeological permits were required for the described study as it relies on already published faunal and dating observations.

### 2. Measuring the rate of spread

It is possible to estimate the Neolithic spread rate (i.e., the front speed) from the archaeological dates by considering several spatial points (e.g. the oldest sites in the database) as possible sources for the Neolithic range expansion, and fitting a linear regression for each possible source. The source with the highest correlation coefficient (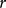) will be the most probable source and will yield the best estimate for the Neolithic rate of spread [1], [2]. The relevant linear regressions are computed as follows. For each possible source (a site in the database) and a calibrated date of this site, we compute the time interval between the mean of this date and the mean calibrated date of each of the other sites, as well as the geographical distance between the presumed source and each site. We first compute distances as great-circle distances, that is, the shortest path joining both sites on the Earth surface, if considered a sphere. The great-circle distance between two locations 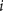 and 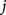 can be calculated from their geographical coordinates (latitude 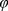 and longitude 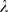) using the Haversine equation [44] and the average value of the Earth radius 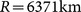 as 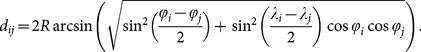
(1)


The accuracy of time intervals is affected by dating and calibrating errors, the fact that only a small fraction of the archaeological sites has been discovered, etc. In contrast, distances between sites are in principle known with a high degree of precision. Therefore, the front speed is computed by plotting the time intervals versus distances and performing a linear regression [45] (and refs therein). The speed is then calculated from the slope as follows:
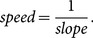
(2)


Applying error propagation [46], the standard error for the speed is obtained from the slope and its standard error with the following expression
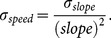
(3)


Since the number of dates available for the spread of the Neolithic in southern Africa (Table 1) is small (as compared, e.g., to the 735 dates for Europe [2]), we will compute the 80% confidence-level interval for the speed from each regression, i.e. 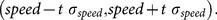
(4)


This range is centered around the speed estimated from Eq. (2) and has an 80% probability of containing the true value of the front speed, provided that we use the value of 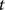 from the tables of Student's *t*-distribution for *N*-2 degrees of freedom, where *N* is the number of data pairs used (each pair being a time interval and its distance) and a confidence level of 80% (this value of 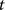 is usually referred to as *t*_.90_) [47], [48].

As explained above, the reason why we plot time intervals versus distances (not distances versus time intervals) is that dates are affected by various errors, whereas distances between sites are in principle known with precision. However, such distances are not absolutely precise because landforms such as seas, mountains, etc. can act as obstacles or introduce local deviations to the speed of the wave of advance. For this reason, as in previous work [2], below we will also introduce shortest-path distances and compare the results to those using great-circle distances.

## Results

Fig. 1 is an interpolation map of mean calibrated dates. We see that the oldest sites with domestic fauna are located at the northwest. The Neolithic wave of advance propagated southward at a fairly constant rate on average, although there are relevant differences for some nearby sites (e.g., the dates of sites 11 and 12 are quite late, as compared to those of sites 13–14 and 16–17, Table 1).

### 1. Great-circle approach

In order to estimate the spread rate of this Neolithic front, in Fig. 2 we plot dates versus distances, both of them computed from the oldest site, which we assume to be the source of the Neolithic wave of advance (this site is Leopard Cave, Namibia, labeled as 4 in Fig. 1 and Table 1; its oldest calibrated date is 280 BC). The horizontal axis in Fig. 2 corresponds to great-circle distances (see Materials and methods), computed between the presumed source (site 4) and each site (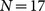, see Table 1). The vertical axis in Fig. 2 is the time difference between the oldest date of the presumed source (280 BC, see above) and each date in Table 1. Using other sites as possible sources yielded lower values for the correlation coefficient 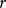
(not only for the regression in Fig. 2, but also for all other regressions reported in this paper).

The linear regression in Fig. 2 yields a high value of the correlation coefficient (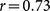), which confirms that the speed was fairly constant, and a speed range of 1.4–2.8 km/yr (80% confidence-level interval).

The speed of the European Neolithic transition has been previously computed using the same method (i.e., regressions of calibrated dates versus great-circle distances). It is rather slower, namely 0.9–1.0 km/yr [2] (see protocol S1, Fig. 2c therein). Thus, the spread of herding in southern Africa (2.1±0.7 km/yr using great circles) was substantially faster than the spread of farming and stockbreeding in Europe (∼1.0 km/yr using the same approach).

Russell [21] has performed very interesting analyses by following a methodology very similar to ours. Indeed, she also plotted calibrated dates versus great-circle distances. By carefully selecting the sites according to their reliability, she was able to obtain high correlation coefficients in some cases (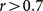). However, the spread rates estimated by Russell [21] are slower than ours. This is due to the fact that she considered a substantially smaller region. For example, consider the 4 sites in her group 1 (this is her most reliable group, i.e., herder sites with direct Accelerator Mass Spectrometry determinations on sheep bone). Those 4 sites correspond to our sites 8, 9, 11 and 12 (Table 1). They are all located at the right of Fig. 2, which corresponds to part of the region considered here (note from Fig. 2 that the distance range of those 4 sites is about 500 km, whereas our data extend over about 1600 km, i.e. our distance range is about 3 times larger than for the database used by Russell [21]). Noteworthy, the dates of these 4 sites used by Russell [21] are very similar to ours, so there is no inconsistency between her data and ours. Note also that if we used only those sites (numbers 8, 9, 11 and 12) in Fig. 2, we would obtain a much steeper linear fit, and thus a slower speed, in agreement with the results by Russell [21]. As a first step, Russell's [21] study was very useful, and our analysis offers an improvement upon it by including a substantially larger region and dataset which lead to more robust estimations of the spread rate of non-agricultural herding in southern-most Africa.

### 2. Shortest-path approach

We may note in Fig. 1 that most sites are located in the western part and near the Atlantic Ocean, whereas there are huge inland areas without any known sites. For this reason, we cannot guarantee that the Neolithic front spread across those huge areas. In case it did not, clearly the site located at the lower right in the map (site 5 in Fig. 1, i.e. site Likoaeng in Table 1) might have introduced a substantial bias in the speed range estimated above. In fact, for many years some scholars have suggested that the spread of the southern African Neolithic proceeded southward along the Atlantic coast to southern-most Africa (e.g., site 17 in Fig. 1), and then eastward [15], [16]. In order to see to what extent this possibility might affect our conclusions, we repeat the regression in Fig. 2 but applying a shortest-path approach [2] for site number 5 (Fig. 1). In this case, the distance from site 4 in Fig. 1 (the presumed source of the Neolithic in the region) to site 5 is computed not as the great-circle distance between both sites (as in Fig. 2), but as the sum of the great-circle distance from site 4 (the source) to site 17 (lower left in Fig. 1) plus the great-circle distance from site 17 to site 5. Such a distance was called a shortest-path distance (computed along the presumed front propagation direction) and applied to the European Neolithic in Pinhasi et al. [2]. Then the distance for site 5 is larger and the correlation coefficient increases (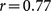, with again 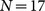 dates), as was to be expected from Fig. 2. We think that this increase in the value of *r* can be interpreted as giving quantitative support to the proposal that the southern African Neolithic front propagated southward mainly along the Atlantic coast and later eastward [15], [16]. However, we would like to stress that more sites should be dated before this scenario is given more weight (see Fig. 1). Crucially for our purposes here, the speed range obtained from the shortest-path approach is 1.7–3.1 km/yr (80% confidence-level interval), almost the same as that obtained above from the great-circle approach (1.4–2.8 km/yr, Fig. 2) and much faster than that of the European Neolithic transition (1.1–1.2 km/yr using calibrated dates versus shortest paths, and 0.9–1.0 km/yr using calibrated dates versus great circles, (see [2] and protocol S1 therein).

As a further check of our estimations of the speed, we can also take into account that there are several nearby western sites on the left in Fig. 1, so keeping only the oldest ones might perhaps have an effect. Therefore, we leave out three sites whose dates are more than 500 yr younger than other sites at essentially the same distance from the source (these are sites 7, 11 and 12, see Fig. 2). Then (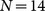), using again the shortest-path approach, the correlation coefficient increases substantially (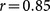), as expected, but the speed range (2.0–3.3 km/yr) remains similar and, remarkably, much faster than the speed of the European Neolithic front (1.1–1.2 km/yr, using also shortest paths).

**Figure 3:**
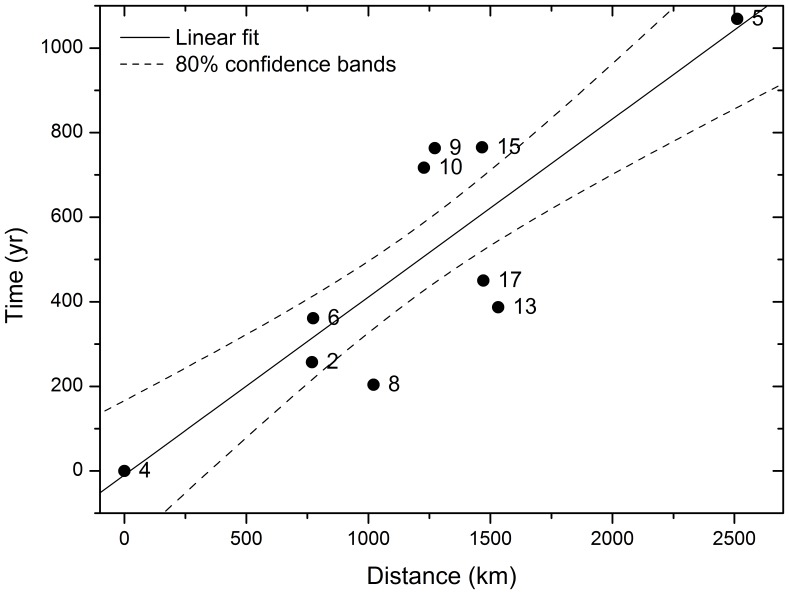
Linear regression fit to determine the speed of the Neolithic fronts using oldest dates and shortest-path routes. Symbols correspond to the 10 dates from table 1 selected as regionally oldest. Time and distances are computed from the oldest site (labelled 4 in the Fig. and in table 1) using calibrated dates. Distances are computed using great-circle routes except for site Likoaeng (labelled 5), for which we used a shortest-path route through site Hawston (labelled 17, see Fig. 1). The solid line corresponds to the linear regression fit and the dashed lines are the 80% confidence bands. The 80% confidence level range obtained for the front speed is 1.6–3.1 km/yr with a correlation coefficient 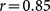.

Finally, in addition to the two checks presented above, let us also consider only the oldest date for those sites with more than one date in Table 1. The corresponding regression is shown in Fig. 3 (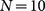 dates) and yields again very similar results (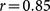 and the speed range 1.6–3.1 km/yr, 80% confidence-level interval).

The important point is that all of these analyses lead to a global, shortest-path range of 1.6–3.3 km/yr, which is very similar to that determined by using all dates and great circles (1.4–2.8 km/yr, Fig. 2). Combining the shortest-path and great-circle ranges, we can thus safely conclude that the spread rate of the southern African Neolithic was 1.4–3.3 km/yr (or 2.4±1.0 km/yr), about twice faster than that of the European Neolithic (0.9–1.2 km/yr, again using calibrated dates and combining the great-circle and shortest-path ranges). The error range for Europe is smaller because there are substantially more dated sites in Europe [2] than in southern Africa.

In subsection 4 below we show that excluding far-inland sites does not change the main results and conclusions of the present paper.

### 3. Demic versus cultural diffusion

As recalled above, the speed of the European Neolithic transition has been previously estimated as 0.9–1.2 km/yr using calibrated dates (this is almost the same as the uncalibrated range, 1.0–1.3 km/yr [2], and both ranges were combined for the European Neolithic in ref [8], but here we use only the calibrated range—both for the European and for the southern African Neolithic—because it is more accurate and also because most authors working on southern Africa use calibrated dates). Such a range implies that the European Neolithic transition was mainly demic, specifically about 60% demic and 40% cultural, according to a recent wave of advance model that unifies demic diffusion and cultural transmission [8]. Demic diffusion alone would have led to a speed slower than the observed range of 0.9–1.2 km/yr [8] (see therein Fig. 1 for 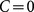), so cultural transmission was responsible for the difference between the observed rate and the slower one predicted by purely demic diffusion. In the previous subsections we have found that the speed of the southern African Neolithic transition was 1.4–3.3 km/yr, substantially faster than the European one. What are the demic and cultural percentages for the southern African Neolithic? This is the question we tackle in this section.

First of all, because many sites are located near the coast (Fig. 1) we might be tempted to use a one-dimensional model (along the coast) rather than the two-dimensional model applied to the European Neolithic transition [8]. However, we note from Fig. 1 that about one third of sites are located at distances larger than 100 km from the coast. Therefore, it does not seem reasonable to use a one-dimensional model for the whole process. A one-dimensional process might be perhaps justified only for a local region (sites 7–14 in Fig. 1) but this would introduce a huge error in the speed (similarly to what happened for the local region considered by Russell [21], see above). Moreover, additional dated sites are necessary before such a coastal route can be justified. For these reasons, it seems more reasonable to consider the whole region in Fig. 1 (so that we can apply the speed ranges calculated above) and use a two-dimensional model. A one-dimensional model would imply the assumption that all herders lived on the coast or very close to it, and this is clearly inconsistent with a substantial part of the sites (Fig. 1).

A two-dimensional model that combines demic diffusion and cultural transmission theory [49] has been presented recently, and leads to the following speed of Neolithic fronts [8]



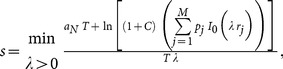
(5)


where 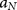 is the reproduction rate (initial growth rate) of the Neolithic population, *T* is its generation time (mean age difference between parents and their children), and *C* is the intensity of cultural transmission (see ref [8] for the complete derivation and details). This model also takes into account that newborn humans need to spend some time with their parents before they can survive on their own and migrate (cohabitation effect) and the detailed dependence of the migration probability as a function of distance [8]. The latter effect is taken into account in Eq. (5) by means of *p_j_*, defined as the probability of the Neolithic individuals (herders in our case) to disperse a distance 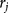 (*j = 1,2,…,M*). Finally 
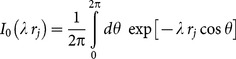
 is the modified Bessel function of the first kind and order zero.

It is worth mentioning that a simpler model, which is Fisher's wave-of-advance model generalized to include cultural transmission, was also presented [8] (see Eqs. (S10)–(S11) and Supp. Info. therein) but it is less accurate, so we prefer to use Eq. (5) instead. A simple way to see the limitations of the generalized Fisher model is to note that it predicts an infinite speed in the limit 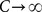, whereas Eq. (5) predicts a finite result, namely the maximum dispersal distance divided by the generation time, which is intuitively very reasonable. This point was checked numerically in [8] (below Eq. S11 therein) where additional explanations and a detailed comparison of both models can be also found.

In order to apply equation (5), we need the values of the parameters 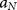, *T*, *p_j_*, *r_j_* and *C*. We discuss them in turn. Firstly we deal with the reproductive parameters 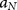 and *T*. It is well-known that 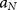 is the reproduction rate in the ‘growing fringe’ at the periphery of the expanding population range (front leading edge) [6], and should thus be estimated for small populations that settled in empty space (then the population density is far from saturation, and population growth is still exponential). Moreover, the reproductive behavior of farmers and herders does not seem to be substantially different [8]. Therefore, for 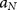 we use the range 0.023 yr
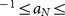
0.033 yr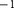 (as obtained from ethnographic and archaeological observations of several small preindustrial populations that settled in empty space), and for the generation time *T* we use 29 yr 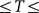
35 yr (also the observed range for preindustrial populations, see Supp. Info. in ref [8] for details on both parameter ranges).

Secondly, the dispersal behavior of the population is described by the following probabilities and distances, which were obtained from 4,483 observations of parent-offspring birthplace distances for populations of herders (ref 46: p. 208), {*p_j_*}  =  {0.67; 0.05; 0.04; 0.07; 0.08; 0.04; 0.05} and 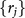  =  {0.5; 3; 7.5; 15; 25; 35; 95} km. Here we have used the average distance for each interval of the reported histogram and made sure that the mean distance agrees with that reported by Mehrai [50]. This approach to compute dispersal kernels was already applied to the Issocongos by Isern et al. [51], where purely demic models of farmers were analyzed. In contrast, in the present paper we consider demic-cultural models of herders.

Thirdly, the cultural effect is witnessed in Eq. (5) by the intensity of cultural transmission *C.* This parameter *C* is the average number of hunter-gatherers converted into herding by each herder per generation at the leading edge of the wave of advance, i.e. when the first herders arrive and their population density is still much lower than that of hunter-gatherers [8]. Thus,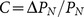, where 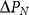 stands for the number of hunter-gatherers converted into herding, and 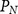 for the initial number of herders [8]. In order to estimate a range for *C*, let us first consider the following example. By the early twentieth century, a German immigrant landholder contacted a small group of Ache hunter-gatherers in Paraguay, converted them into herding, and they lived in his ranch for years [52]. By comparing to other contacted groups that are quantified by Hill and Hurtado [52], a reasonable estimate of the size of this small group is 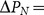 6–15 individuals. Since the immigrant landholder had arrived on his own (he later married a hunter-gatherer woman), 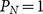 and therefore 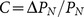 is in the range 6
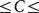
15. In the second half of the twentieth century there are more detailed population data for another ranch (that became a reservation), and the same calculation as above yields 5.5
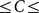
10.9. Thus the overall range for the intensity of cultural transmission is 5.5
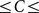
15.

**Figure 4:**
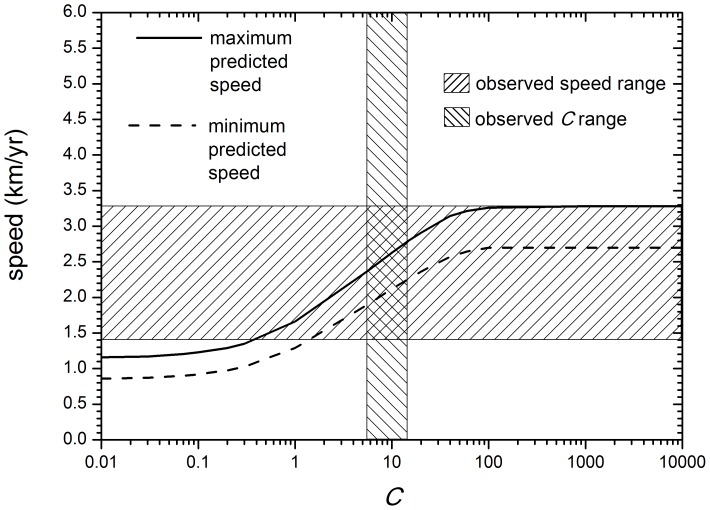
The full curve is the maximum speed predicted by Eq. (5), the dashed curve is the minimum speed predicted by Eq. (5), the horizontal hatched rectangle is the observed speed range, and the vertical hatched rectangle is the observed range of cultural transmission intensity *C*. The purely demic model corresponds to 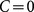 (i.e., no conversion of hunter-gatherers into herding).

Using the parameter's values in the previous paragraph into Eq. (5) leads to Fig. 4, which displays the predicted Neolithic front speed range as a function of the intensity of cultural transmission 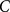. The full curve in Fig. 4 corresponds to the maximum speed predicted by the model, i.e. from Eq. (5) with 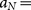
0.033 yr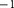 (maximum reproduction rate) and 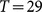 yr (minimum generation time). The reason why the maximum speed corresponds to the minimum generation time is that the latter is the time interval between subsequent dispersal events in the model (see e.g. ref [8]). The dashed curve in Fig. 4 corresponds to the minimum predicted speed (i.e., for the minimum reproduction rate, 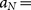
0.023 yr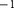, and the maximum generation time, 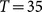 yr).

The observed range for the intensity *C* of cultural transmission (5.5–15, see above) corresponds to the vertical hatched rectangle in Fig. 4. For this observed range of *C*, we note that the predicted speed range (i.e., that between the dashed and full curves in Fig. 4) is consistent with the observed speed range of the Neolithic front of herding in southern Africa (horizontal rectangle, namely 1.4–3.3 km/yr, as derived from the linear regressions in the previous sections).

**Figure 5:**
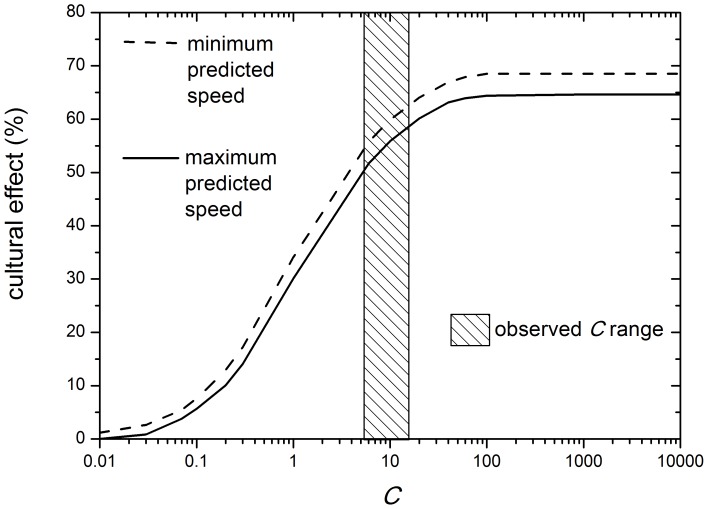
Effect of cultural transmission on the front speed, defined as the speed with cultural transmission (i.e., that plotted in Fig. 4 for the value of *C* considered) minus the speed without cultural transmission (i.e., that plotted in Fig. 4 for 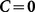), divided by the former and multiplied by 100. The full curve has been obtained using the speed as a function of *C* given by the full curve in Fig. 4. The dashed curve has been obtained using the dashed curve in Fig. 4. The vertical rectangle is the observed range of cultural transmission intensity *C*. The purely demic model (cultural effect of 0%) corresponds to 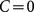 (i.e., no conversion of hunter-gatherers into herding).

In order to estimate the importance of cultural versus demic diffusion, we follow the approach previously applied to the transition to farming [8] but here we deal with herding (rather than farming) Neolithic fronts. Thus, in Fig. 5 we plot the cultural effect (in %), defined as the speed with cultural transmission (i.e., that from one of the two curves in Fig. 4 for the value of *C* considered) minus the speed without cultural transmission (i.e., that from the same curve in Fig. 4 for 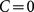), divided by the former and multiplied by 100.

According to Fig. 5, the observed dispersal kernel of herding populations and the observed intensity of conversion from hunting-gathering into herding (hatched rectangle) lead to the conclusion that the cultural effect on the Neolithic wave of advance was 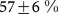. This is noticeably different than the Neolithic transition in Europe, where the cultural effect was estimated as 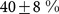 (8). Therefore we conclude that the Neolithic front of the herding Neolithic in southern Africa was mainly cultural, whereas the Neolithic front of the farming and stockbreeding Neolithic in Europe was mainly demic.

Genetic research, although based only on extant populations, has introduced important insights into the spread of pastoralism in southern Africa (for instance, refs 30–33). Observations on Y-chromosomes, mitochondrial DNA and presence of lactase persistence genes suggest that a migration of non-Bantu-speakers to southern Africa took place between 2,700 and 1,200 years ago, a timing that is in accord with the absolute chronology discussed here (Table 1 and Fig. 1). Indeed, our model does accommodate some level, although minor, of demic processes as part of the southern African Neolithic transition. Improving the chronological resolution of genetic reconstructions and comparability of the data sets ought to be achieved in future with additional and geographically overlapping genetic and archaeological data.

### 4. Effect of far-inland sites

In Fig. 1 we see that almost all of the sites are located near the west coast. In the absence of information from the intervening areas, it is reasonable to suspect that the two far-inland sites (dates 1 and 2 in northern Botswana and date 5 in Lesotho) might perhaps not be representative of the main expansion process. In this subsection we estimate the distortion introduced by those dates by repeating the calculations without them. Note that, in doing so, the great-circle and the shortest-path approaches will be identical because date 5 (Fig. 1) will not be used in the new analysis. In addition, because we are working with fewer dates, we can expect the statistics to be poorer.

If we remove far-inland dates (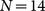) we obtain 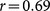 (versus 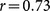 with 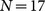, Fig. 2) but the 80% confidence-level interval is 1.4–3.4 km/yr (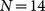), consistent with the result with all sites (1.4–2.8 km/yr with 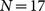, Fig. 2).

We can further refine the analysis but still use a reasonable number of dates, e.g. by excluding also dates 7, 11 and 12 (which are>500 yr younger than other dates at similar distances), as in Sec. 3.2. Then 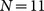, 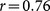 (versus 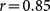 for 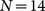 in Sec. 3.2) and the speed range is 1.8–4.1 km/yr, again consistent with the corresponding result in Sec. 3.2 (2.0–3.3 km/yr).

Neglecting far-inland sites therefore yields the overall range 1.4–4.1 km/yr. This would only change the upper side of the horizontal hatched rectangle in Fig. 4 (i.e. the observed speed range, which is 1.4–3.3 km/yr in Fig. 4, from the regressions in Sec. 3.2). But in spite of this minor change in Fig. 4, obviously Fig. 5 would remain exactly the same (because for 5.5
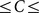
15, the speed range predicted by the model, i.e. that between the two curves in Fig. 4, would still be within the horizontal hatched rectangle, 1.4–4.1 km/yr). Thus the implied cultural effect would be 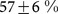, i.e. exactly the same as in the previous subsection. Therefore, far-inland sites do not affect the results of our paper.

## Conclusions

In the present paper we have found that the Neolithic transition in southern Africa spread at a rate of 2.4±1.0 km/yr. This is substantially faster than in Europe (1.0±0.2 km/yr). We conclude that the well-known European value (∼1 km/yr) is not a universal feature of Neolithic transitions.

We have also found that the speed of the Neolithic spread in southern Africa implies that, in this case, demic diffusion was *less* important than cultural diffusion. This is in sharp contrast with the spread of the European Neolithic transition where demic diffusion was *more* important than cultural diffusion [8]. Thus the primacy of demic over cultural diffusion is not a general law of Neolithic transitions around the world.

According to the model, the three crucial features are reproduction, dispersal and cultural transmission. It is interesting to establish their relative importance on the question posed, namely the primacy of demic or cultural diffusion. The reproductive behavior of farmers and herders does not seem to be substantially different [8]. Similarly, differences in their dispersal behavior are not important because, if instead of dispersal kernels of herders (present paper) we used dispersal kernels of farmers [8] we would again obtain a substantially higher cultural effect for a fast front (e.g. 2.4 km/yr) than for a slow one (e.g. 1.0 km/yr). Thus, the fastness of the Neolithic transition in southern Africa (as compared to Europe) is probably not due to differences in dispersal or reproductive behavior between herders and agro-pastoralists, but most likely to a higher ease for hunter-gatherers to learn herding (as compared to farming).

For the purpose of improving our understanding of Neolithic transitions in other areas (e.g., [53]–[55]), high-quality data will be necessary. Doing so will allow to determine (i) whether *demic* diffusion dominated all (or most of) Neolithic transitions into *farming*, as for the European Neolithic transition [8]; (ii) whether *cultural* diffusion dominated all (or most of) Neolithic transitions into *herding*/*pastoralism*, as for southern Africa (present paper).

The model derived in ref [8] and applied here to a specific case study could be used in future work to determine the importance of demic and cultural diffusion in other interesting examples, such as the spread of Iron Age from western to southern Africa [55] and the spread of horses in North America [56].
